# Editorial: Insight in cancer genetics: 2022

**DOI:** 10.3389/fonc.2022.988310

**Published:** 2022-07-26

**Authors:** Chiara Naro, Heather E. Cunliffe, Claudio Sette

**Affiliations:** ^1^ Department of Neuroscience, Section of Human Anatomy, University of the Sacred Hearth, Rome, Italy; ^2^ Gemelli Science and Technology Park (GSTeP)-Organoids Research Core Facility, Fondazione Policlinico Agostino Gemelli IRCCS, Rome, Italy; ^3^ Department of Pathology, Dunedin School of Medicine, University of Otago, Dunedin, New Zealand

**Keywords:** cancer genetics, next-generation sequencing, CRISPR-Cas9, personalized medicine, anti-cancer therapies

Tumorigenesis is a multistep process, driving normal cells to the progressive acquisition of neoplastic features (1). Development of hallmarks of cancer is induced by the accumulation of genetic defects that alter gene expression patterns that maintain cellular homeostasis (1). Human cancers have been recognized as genetic diseases since 1970 (2–4). Since then, huge efforts have been made to identify the genetic alterations implicated in tumour pathogenesis. This has propelled development of targeted therapies and tools for early diagnosis and prevention, leading to great improvements in cancer care. Hereditary breast cancer and the *BRCA1* and *BRCA2* genes provide a paradigm of the tangible improvements that cancer genetic studies produce in the clinical practice. Identification of pathogenic alterations of these loci in tumors has led to the implementation of preventive strategies based on genetic screening (5, 6). In addition, the characterization of the role of BRCA1/2 proteins in DNA repair has facilitated the development of targeted therapeutic approaches, such as treatment with PARP inhibitors (5, 6). Importantly, several pre-clinical and clinical studies have shown that benefits of these targeted therapies are extended to other cancers that display a so-called BRCANESS phenotype, due to sporadic and germline mutations in *BRCA1* and *BRAC2* or other genes involved in DNA repair (7, 8). These observations witness the great relevance of cancer genetic studies for development of novel and more efficacious therapeutic strategies.

In the last decade, the advent of the next-generation sequencing (NGS) and CRISPR-Cas9 technologies have led to breakthroughs in the field of cancer genetics. Massively parallel sequencing is allowing cost- and time-effective genome-wide analyses of DNA samples from large cohorts of patients. Moreover, advanced computational analyses integrating genomic and clinical data enables identification of cancer-driver mutations and biomarkers of drug response and resistance (9). CRISPR-Cas9 gene-editing tools are allowing rapid and precise targeted modification of genomes, which can be employed in medium/large-scale functional genetic studies using primary or immortalized cell-lines, patient-derived xenografts and organoid cultures, as well as genetically engineered animal models (10, 11). The articles included in this Research Topic “Insight in Cancer Genetics: 2022” have exploited these novel technologies to search for the genetic determinants of tumor development and progression of four different cancer types: breast cancer, acute leukemia, colorectal adenocarcinoma and hepatocellular carcinoma.

Tao et al. applied NGS technology to investigate the prevalence and spectrum of chromosomal aberrations and mutations occurring in receptor tyrosine kinases (RTKs) among a large cohort of Chinese breast cancers patients. This study revealed a prevalence of RTK fusion events of 1.875%, higher than previously observed in non-Chinese patients from the MSKCC (12) and TCGA database (13). Moreover, they observed a negative correlation between the abundance of RTK fusions and the tumoral mutational burden, which is suggestive of an oncogenic driver activity for these chromosomal aberrations in some patients. Given the remarkable efficacy that targeted inhibitors against RTK gene-fusions have shown in select haematological and solid tumors in patients (14), this study supports the relevance of genomic analysis for the identification of breast cancer patients that might also benefit from such targeted therapeutic approaches.

The studies from Lee et al. and Li et al. illustrate the contribution provided by transcriptome profiling and genome sequencing to the identification of genetic determinants of cancer onset and progression. Lee et al. employed RNA-sequencing analyses of 12 cases of BCR-ABL1-positive B-lymphoblastic leukemia (B-ALL), acute myeloid leukemia (AML), and mixed-phenotype acute leukemia (MPAL), detecting DNA mutations and gene-fusions, and definition of splice-sites involved in these latter chromosomal alterations. Moreover, analysis of the differentially expressed genes between AML and B-ALL allowed the development of two distinct algorithms for differential diagnosis of these disease subtypes. Results from this study support the idea that the significant cost-reduction observed for NGS experiments in the last decade will soon usher in routine use of these methodologies in the clinic, enabling more precise cancer diagnosis, classification and treatment planning. Li et al. showed that the potential of NGS approaches to decipher cancer genetics is not limited to their ability to identify mutation and chromosomal aberrations. Indeed, new prognostic markers and therapeutic targets might also be revealed by functional annotation of genomic and transcriptomic data. To identify novel prognostic biomarkers for colorectal adenocarcinoma (CRAC), the authors performed comparative transcriptome analysis of mismatch repair-deficient (dMMR) and -proficient (pMMR) patients, as these latter patients are characterized by worse prognosis and higher metastastic rate (15). They developed a prognostic signature made of seven genes related to glycosylation, named GlycoSig. The signature proved to be valid and robust across multiple datasets, suggesting that it could also represent a valuable therapeutic target for CRAC treatment. Selection of the GlycoSig signature was guided by gene-ontology and pathway enrichment analyses, which retrievied a significant enrichment for this term among the genes differentially expressed between dMMR and pMMR patients. Thus, these three studies show how novel-genetic determinants of cancer transformation, progression and chemoresistance can be unveiled by NGS-studies. However, the clinical translation of these findings requires investigation of the underlying molecular mechanisms in reliable cellular and animal models. Elkhadragy et al. provide an example of how CRISPR-Cas9 technology has facilitated this process. Genome sequencing studies identified *ARID1A* and *AXIN1* among the most frequently mutated genes in human hepatocellular carcinoma (HCC). The authors ablated these two genes, either alone or simultaneously, by using CRISPR-Cas9-guided editing in two porcine HCC cell lines, thereby generating cellular models that recapitulate the loss of function mutations observed in HCC patients. These edited cell lines represent optimal laboratory models to analyse *ARID1A* and *AXIN1* oncogenic activity, both *in-vitro* and in *ex-vivo* xenograft porcine models. This study demonstrates the feasibility of developing clinically relevant experimental models that faithfully recapitulate the genetic alterations found in primary tumors, thus paving the ground for the development of precision medicine approaches.

Collectively, these studies provide remarkable examples of the propulsive force that novel technologies, such as NGS and CRISPR-Cas9, are exerting in cancer genetics discovery research. However, the precise challenge of this new era of oncogenomics is the realization of experimental models that rapidly and effectively allow the translation of tumor molecular-genetic knowledge into clinical decisions. For instance, amelioration and lowering of the costs of spatial- and single-cell genomic technologies will allow discrimination of genetic alterations occurring in cancer cells from those occurring in the tumor microenvironment, and to investigate their reciprocal interaction. Developing and optimizing this workflow is the real prerequisite for the development of personalized- and precision-cancer medicine.

## Author contributions

All authors listed have made a substantial, direct, and intellectual contribution to the work and approved it for publication.

## Funding

This work was supported by grants from the Associazione Italiana Ricerca sul Cancro (IG23416 and MFAG21899) and Breast Cancer Now (Catalyst Grant n. 2018NovPCC1283). Università Cattolica del Sacro Cuore contributed to the funding of this research project and its publication.

## Conflict of interest

The authors declare that the research was conducted in the absence of any commercial or financial relationships that could be construed as a potential conflict of interest.

## Publisher’s note

All claims expressed in this article are solely those of the authors and do not necessarily represent those of their affiliated organizations, or those of the publisher, the editors and the reviewers. Any product that may be evaluated in this article, or claim that may be made by its manufacturer, is not guaranteed or endorsed by the publisher.
